# Multi-Stage Network for Event-Based Video Deblurring with Residual Hint Attention

**DOI:** 10.3390/s23062880

**Published:** 2023-03-07

**Authors:** Jeongmin Kim, Yong Ju Jung

**Affiliations:** School of Computing, Gachon University, Seongnam 13120, Republic of Korea

**Keywords:** event-based vision, video deblurring, deep learning, multi-stage network, attention

## Abstract

Video deblurring aims at removing the motion blur caused by the movement of objects or camera shake. Traditional video deblurring methods have mainly focused on frame-based deblurring, which takes only blurry frames as the input to produce sharp frames. However, frame-based deblurring has shown poor picture quality in challenging cases of video restoration where severely blurred frames are provided as the input. To overcome this issue, recent studies have begun to explore the event-based approach, which uses the event sequence captured by an event camera for motion deblurring. Event cameras have several advantages compared to conventional frame cameras. Among these advantages, event cameras have a low latency in imaging data acquisition (0.001 ms for event cameras vs. 10 ms for frame cameras). Hence, event data can be acquired at a high acquisition rate (up to one microsecond). This means that the event sequence contains more accurate motion information than video frames. Additionally, event data can be acquired with less motion blur. Due to these advantages, the use of event data is highly beneficial for achieving improvements in the quality of deblurred frames. Accordingly, the results of event-based video deblurring are superior to those of frame-based deblurring methods, even for severely blurred video frames. However, the direct use of event data can often generate visual artifacts in the final output frame (e.g., image noise and incorrect textures), because event data intrinsically contain insufficient textures and event noise. To tackle this issue in event-based deblurring, we propose a two-stage coarse-refinement network by adding a frame-based refinement stage that utilizes all the available frames with more abundant textures to further improve the picture quality of the first-stage coarse output. Specifically, a coarse intermediate frame is estimated by performing event-based video deblurring in the first-stage network. A residual hint attention (RHA) module is also proposed to extract useful attention information from the coarse output and all the available frames. This module connects the first and second stages and effectively guides the frame-based refinement of the coarse output. The final deblurred frame is then obtained by refining the coarse output using the residual hint attention and all the available frame information in the second-stage network. We validated the deblurring performance of the proposed network on the GoPro synthetic dataset (33 videos and 4702 frames) and the HQF real dataset (11 videos and 2212 frames). Compared to the state-of-the-art method ($D^{2}$Net), we achieved a performance improvement of 1 dB in PSNR and 0.05 in SSIM on the GoPro dataset, and an improvement of 1.7 dB in PSNR and 0.03 in SSIM on the HQF dataset.

## 1. Introduction

Video deblurring is a computer vision task for obtaining sharp frames by removing the motion blur from blurry frames. A motion blur causes the structure of an object to become blurry and distorts edges in a video frame, thereby lowering the picture quality. A motion blur also hinders the extraction of high-level semantic information from a video, which eventually causes difficulties in video recognition tasks such as object detection or object tracking. Due to these reasons, video deblurring is a low-level vision task with extremely high utilization and significance.

Previous studies mainly focused on frame-based video deblurring [1,2,3,4,5,6,7,8,9,10,11], which involves using only the sequence of blurry frames as an input. Note that each pixel’s intensity values in a video frame are acquired by the sum of incident light from a scene during the exposure time, causing a loss in temporal information within the exposure time. Therefore, the goal of frame-based deblurring is to infer the motion information lost within the exposure time and then remove the blur from the blurry frame. Various video deblurring methods have been studied, ranging from handcrafted methods to deep learning methods using convolutional neural networks, achieving significant progress. However, severely blurred videos, which are challenging cases for restoring the lost motion information, suffer from significant degradation in restoration quality. To overcome the performance limitation of frame-based video deblurring, event-based video deblurring [12,13] has emerged. Event data, which are used as an auxiliary input in event-based video deblurring, are captured from a bio-inspired vision sensor called an event camera [14,15], which better preserves motion information thanks to its high acquisition rate.

An event camera independently and asynchronously records the event data from each pixel. The recorded event data contain four-dimensional values of (*x*, *y*, *t*, *p*), where *x* and *y* represent the location in two-dimensional space; *t* indicates the timestamp at which an event occurred; and *p* is a polarity value indicating the direction of events as determined by the triggering contrast threshold value, which is a camera parameter. The relationship between the triggering contrast threshold value and polarity can be expressed as:

(1) $$p=\left\{\begin{array}{cc} +1, & d\geq c\\ 0, & d\in (-c,c)\\ -1, & d\leq -c \end{array}\right.$$

**Figure 1:**
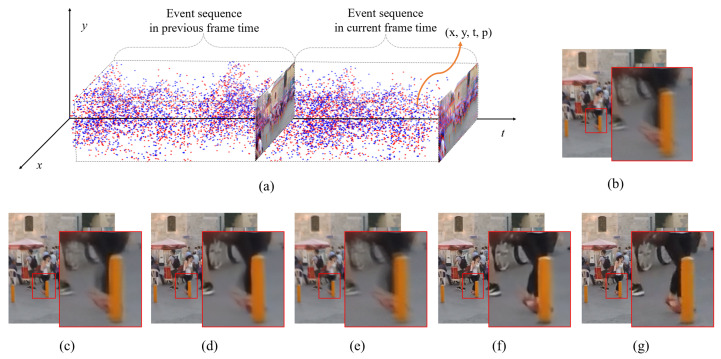
(**a**) Event sequence data. (**b**) Current blurry frame. (**c**) STFAN [5] result. (**d**) CDVD-TSP [8] result. (**e**) ESTRNN [10] result. (**f**) Proposed MEVDNet result. (**g**) Ground truth.

Here, *p* represents the polarity; *c* is the triggering contrast threshold value; and *d* is the log intensity change value. The polarity value is 0 when the magnitude of the log intensity change is smaller than the magnitude of the triggering threshold, which indicates that the event is not activated. The polarity value is unity when the magnitude of the log intensity change is greater than the magnitude of the triggering threshold, and the polarity value is −1 when the magnitude of the log intensity change is smaller than the negative triggering threshold value. Figure 1a shows the forms of event data expressed in a three-dimensional space.

The event sequence data recorded through the above process have several advantages with respect to their low latency, reduced motion blur, high dynamic range, and low power consumption. Due to these advantages, the use of event data has achieved high performance in various vision tasks [16,17,18,19]. Particularly, the low latency and reduced motion blur are highly useful in motion deblurring tasks. Because event data can be obtained at a high acquisition rate (up to one microsecond), the use of event data in motion deblurring enables a significant improvement in picture quality compared with that of frame-based video deblurring. Figure 1 compares the results of frame-based methods (STFAN [5], CDVD-TSP [8], and ESTRNN [10]) and those of the proposed event-based method. As seen from Figure 1c–e, the images deblurred using the frame-based methods still presented distorted edges, and some blur remained. Event-based video deblurring (Figure 1f) provided considerably clearer images than frame-based video deblurring.

Event-based video deblurring can be classified into two approaches: event-main and frame-main. In the event-main method, event data are directly used for the deblurring task. Pan et al. proposed the event double integral (EDI) method [20], which is a handcrafted event-based deblurring method. The EDI method directly computes the intensity residual (i.e., the difference between the blurry frame and the target sharp frame) from event data. The EDI uses the integral of the event sequence data to obtain the intensity residual. LEDVDI [12] improved this technique by applying the EDI to deep learning methods. The event-main method outperformed the frame-based deblurring methods, and outstanding deblurring results were produced, particularly for strongly blurred frames. However, event data often contain acquisition noise and inherently lack texture information, as mentioned in the literature [21]. Hence, the direct use of event data can often generate visual artifacts in the final output image (e.g., image noise and incorrect textures), because event data intrinsically contain insufficient textures and event noise.

Another method, the frame-main method, involves combining event data with the interim process of conventional frame-based deblurring. $D^{2}$Net [13] demonstrated that the picture quality can be improved through the fusion of a high-level frame feature and event feature in a frame-based video deblurring network, which uses optical flow information. Because event data were used as auxiliary data in this approach, incorrect and unstable texture artifacts, which were problematic in the event-main method, were mitigated in the deblurred frames. However, the deblurring was inadequate for the frames presenting a strong motion blur, and the image structure was distorted.

To solve this problem, one possible approach is a hybrid method that combines event-based deblurring with frame-based deblurring in a sequential manner. In this study, we combined the strengths of event-based video deblurring, which shows excellent picture quality regardless of the strength of the blur but has weaknesses in terms of a lack of textures, and frame-based deblurring, which has abundant textures but a relatively low restoration performance. Specifically, to retain the advantages of both approaches, a frame-based method was performed to refine the initial output obtained from an event-based method.

In this study, we propose a two-stage coarse-refinement network for video deblurring. In the first-stage network, a coarse intermediate frame is estimated by performing event-based video deblurring. A residual hint attention (RHA) module is also proposed to extract useful attention information from the coarse output and all the available frames. This module connects the first and second stages and effectively guides the frame-based refinement of the coarse output in the second-stage network. Finally, the second-stage network provides the deblurred frame by refining the coarse output using the residual hint attention and all the available frame information (i.e., the current blurry frame and previously recovered sharp frame).

The contributions of this study are as follows:

- Contrarily to the existing method that uses a single-stage deep learning network, a multi-stage event-based video deblurring network (MEVDNet) is proposed, which estimates a coarse output through event-based video deblurring in the first stage and then refines the coarse output through all the available frame information in the second stage.
- To connect the first and second stages, a RHA module is also proposed to extract useful attention information for the refinement from all the available frame information.
- The performance of the proposed network was verified using the GoPro synthetic dataset [22] and HQF real dataset [23]. Through the comparison experiments, we showed that the proposed method outperformed the state-of-the-art methods in terms of both qualitative and quantitative results.

The rest of the paper is organized as follows. Section 2 introduces the related works on frame-based deblurring and event-based deblurring. Section 3 describes the proposed deep learning model that consists of a two-stage network and a residual hint attention module. Section 4 describes the experimental results that verified the effectiveness of the proposed method. Finally, Section 5 presents the conclusion.

## 2. Related Work

Recent studies have proposed several deep-learning-based methods for image deblurring [24,25,26,27,28,29,30,31,32,33,34,35,36,37,38,39,40]. However, these image deblurring methods do not provide consistency across consecutive frames in a video. Thus, video deblurring methods have been studied to remove the blur from image sequences by additionally using adjacent frames [5,8,10,41].

The related works on video deblurring are largely categorized into frame-based video deblurring [5,8,10] and event-based video deblurring [12,13], as shown in Table 1. In Section 2.1, we describe the frame-based video deblurring methods that estimate sharp frames using only blurry input frames. Recent studies have proposed several deblurring methods based on convolutional neural networks (CNNs), such as STFAN [5], CDVD-TSP [8], and ESTRNN [10]. Here, we summarize the strengths and weaknesses of the deep-learning-based methods. In Section 2.2, we describe the event-based video deblurring methods that perform motion deblurring using event data as an additional input [12,13]. In particular, event-based video deblurring can be further classified into two categories: the frame-main approach and the event-main approach. We also describe the strengths and weaknesses of the event-based deblurring methods.

### 2.1. Frame-Based Video Deblurring

STFAN [5] is a video deblurring method that utilizes the information from previous frames to restore the current blurry frame. Specifically, this method uses a frame alignment technique. The authors of [5] proposed a filter adaptive convolution (FAC) process, which is a kernel prediction network [42,43,44,45] that performs the alignment between previous and current frames. FAC predicts a dynamic filter, which performs the alignment and applies it to high-level features for the video deblurring. Additionally, CDVD-TSP [8] is a video deblurring method that uses the optical flow information for frame alignment. CDVD-TSP predicts the optical flow between adjacent frames before and after the deblurring is applied. Thereafter, a latent sharp frame of a target point is acquired through warping. The final sharp frame is predicted using the latent sharp frame as the input of a convolution neural network. This process is iteratively executed to improve the picture quality of the deblurred frame. ESTRNN [10] also attempts to use adjacent frames and preserves the information of adjacent frames using a recurrent neural network (RNN).

As noted earlier, although deep learning techniques have recently been developed, the frame-based methods show clear limitations for severely blurred videos, which are challenging cases for restoring lost motion information. Hence, these methods often suffer from significant degradation in the restoration quality.

### 2.2. Event-Based Video Deblurring

Event-based deblurring receives event sequence data as an additional input. The limitations of frame-based deblurring are overcome by restoring the lost motion information from the event data [12,13,20]. Event-based video deblurring is further categorized into the frame-main and event-main methods, depending on the role of event data in the deblurring.

The frame-main method involves using event data as an auxiliary input in the deblurring process [13]. This method maintains the structure of a conventional frame-based video deblurring network, which receives blurry frames and then fuses the auxiliary information extracted from the event data. $D^{2}$Net [13] involves reweighing high-level features with event features during frame-based video deblurring. This approach has the advantage of being easy to apply to the framework of frame-based deblurring, which has already been extensively studied. However, it also entails the disadvantage of a poor restoration performance for highly blurry frames, because it is essentially derived from frame-based deblurring.

The event-main method involves directly using event data in the prediction of a motion blur [12,20]. The EDI [20] is a handcrafted event-based deblurring method designed on the basis that event data contain more accurate motion information than intensity frames. Specifically, the intensity residual, which is the difference between the input blur frame and target sharp frame, is predicted using event sequence data. The occurrence of an event in an event camera indicates that a log intensity change exceeding the triggering contrast threshold has occurred at a certain pixel location [20]; that is, a log intensity change is recorded as a polarity value. Hence, the log intensity change can be approximated through the multiplication of the triggering contrast threshold and polarity values. The EDI thus predicts the intensity residual through the integral of the log intensity changes and estimates the sharp frame by adding it to the blurry frame. The EDI, however, applies the same triggering threshold to all pixels. Accordingly, sensor noise has a significant impact, and the results are substantially affected by the given triggering threshold value [12]. The blur is not completely removed when the triggering contrast threshold is low, whereas many visual artifacts are generated due to excessive blur removal when the threshold is high. Nonetheless, the EDI is significant in that event data are directly associated with a motion blur in blurry frames.

LEDVDI [12] has improved deblurring performance by implementing the EDI through a deep learning model. Specifically, LEDVDI extracts the features from event data through an event feature extraction module consisting of convolutional layers. The intensity residual is then predicted based on the extracted event feature. In the event feature extraction module, the triggering contrast threshold is predicted for each pixel using the dynamic filter suggested in STFAN [5].

LEDVDI resolves the problem of the EDI caused by the single triggering threshold value. Additionally, by utilizing previously recovered sharp frames, LEDVDI enhances the picture quality of video deblurring. As a result, this method can provide a high quality of video deblurring regardless of the strength of motion blur.

However, the weakness of this method is that unstable textures or image noise (e.g., stain noise) may occur in the final images, because event data contain insufficient textures and a significant amount of acquisition noise.

## 3. Method

**Figure 2:**
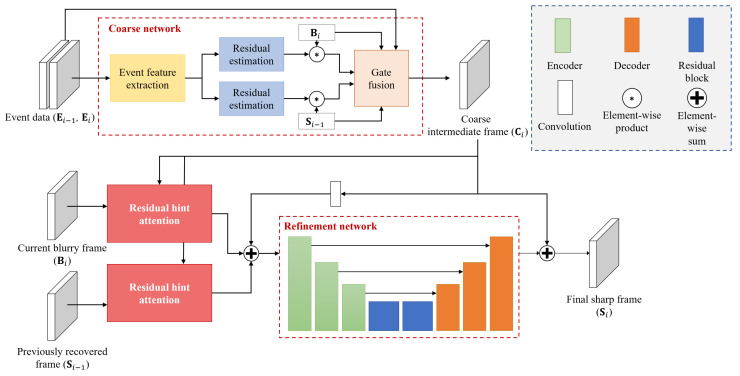
Overall structure of the proposed multi-stage event-based video deblurring network with residual hint attention (MEVDNet).

The proposed end-to-end deep learning model is a multi-stage event-based video deblurring network with residual hint attention (MEVDNet). Figure 2 shows the overall architecture of MEVDNet, consisting of a coarse network in the first stage and a refinement network in the second stage. The coarse network executes event-based video deblurring to generate a coarse intermediate frame using only the event sequence. Two residual hint attention (RHA) modules are located between the coarse network and the refinement network. In this module, attention information that is useful for the refinement is extracted from the coarse frame and two intensity frames (i.e., the current blurry frame $B_{i}$ and previously recovered sharp frame $S_{i-1}$), respectively. In the refinement network, image-based refinement is performed using the coarse frame, which is the output of the coarse network, and two attention features extracted from RHA, and then the final sharp frame is output.

Note that the original event sequence is temporally aggregated, as reported in previous studies [12,46]. Temporally aggregated event data are used as an input for our MEVDNet. Specifically, the temporally aggregated event data are configured by dividing the exposure time during which a blurry frame is captured into *m* temporal blocks, and then the event data for each block are aggregated with respect to the time axis. Similarly to a previous study [12], *m* was set to 18 to train and test the proposed deep learning model.

### 3.1. Stage 1: Coarse Network

In the coarse network, which is the first stage of MEVDNet, a pseudo sharp frame is output through event-based video deblurring. As mentioned earlier, event-based video deblurring using the event-main method can provide clear deblurring results even for severely blurred videos, thus exhibiting outstanding performance for restoring image structures. MEVDNet adopts the method of LEDVDI [12] for a coarse network. In this section, the modules of the coarse network adopted by LEDVDI and the processing flow are briefly described (see the Supplementary Materials for the detailed architecture of the coarse network).

The coarse network receives a blurry frame at the current *i* time ($B_{i}$), a previously recovered sharp frame ($S_{i-1}$), and two aggregated event data ($E_{i-1},E_{i}$) at the previous i−1 time and current *i* time as an input. First, $E_{i-1}$ and $E_{i}$ are concatenated and delivered to the event feature extraction module.

The extracted event features are then used as the input for the residual estimation modules with a two-branch structure. One branch is for the current blurry frame $B_{i}$, while the other branch is for the previously recovered sharp frame $S_{i-1}$. Each residual estimation module consists of two convolution layers and two residual blocks [12]. The residual estimation module returns the intensity residual for each frame image (i.e., $B_{i}$ or $S_{i-1}$). The acquired intensity residuals are multiplied with each input frame to make two latent sharp frames.

Finally, the two latent sharp frames (${\mathrm{LS}}^{\left(1\right)},{\mathrm{LS}}^{\left(2\right)}$) obtained, respectively, from $B_{i}$ and $S_{i-1}$ are fused to output the coarse frame ($C_{i}$) through the gate fusion module [12]. Specifically, the gate fusion module receives $S_{i-1},B_{i},E_{i-1},E_{i}$, and two latent sharp frames (${\mathrm{LS}}^{\left(1\right)},{\mathrm{LS}}^{\left(2\right)}$) as the input. All inputs are concatenated, and a two-channel weight map (W) is returned after passing through three 3D convolution layers and a softmax layer, as follows:

(2) $$W=softmax(Conv3D\left(E_{i},E_{i-1},B_{i},S_{i-1},{\mathrm{LS}}_{i}^{\left(1\right)},{\mathrm{LS}}_{i}^{\left(2\right)}\right)).$$

Using each channel of the obtained weight map to take a weighted sum of the two latent sharp frames (i.e.,${\mathrm{LS}}^{\left(1\right)}$ and ${\mathrm{LS}}^{\left(2\right)}$), the coarse frame $C_{i}$ is generated.

(3) $$C_{i}=W^{\left(1\right)}{\mathrm{LS}}_{i}^{\left(1\right)}+W^{\left(2\right)}{\mathrm{LS}}_{i}^{\left(2\right)}.$$

### 3.2. Residual Hint Attention (RHA)

**Figure 3:**
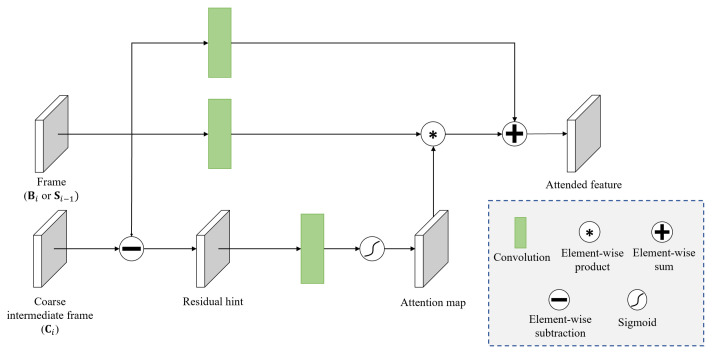
Structure of the residual hint attention (RHA) module.

The attention mechanisms in deep learning models have demonstrated performance improvements in previous studies for various vision tasks [47,48,49,50]. In the proposed method, the RHA module fulfills the role of extracting useful information for refining the coarse frame ($C_{i}$) obtained in the first stage. The structure of the RHA module is illustrated in Figure 3.

The RHA module receives an input frame (i.e., $B_{i}$ or $S_{i-1}$) and the coarse frame ($C_{i}$). Note that, as shown in Figure 2, this process is executed separately in two branches for the current blurry input frame ($B_{i}$) and the previously recovered sharp frame ($S_{i-1}$). The equations below represent the process of the RHA module for the current blurry frame ($B_{i}$).

When the input $B_{i}$ is given, the residual hint ($R_{b}$) is computed. The residual hint can be obtained from the difference between $B_{i}$ and $C_{i}$, as follows:

(4) $$R_{b}=B_{i}-C_{i}.$$

Here, a large value of $R_{b}$ implies that the change in the difference between $B_{i}$ and $C_{i}$ is large, while a small $R_{b}$ value implies that the change is small. The attention map $M_{b}$ is obtained by performing the spatial attention SA() operation consisting of a 3 × 3 convolution and sigmoid for $R_{b}$.

(5) $$M_{b}=SA\left(R_{b}\right).$$

The attention map preserves important information according to the residual hint, while ignoring unnecessary information. The attended feature map ($A_{b}$) is then obtained by determining the element-wise product of the attention map and the 3 × 3 convolution operation for the frame $B_{i}$.

(6) $$A_{b}=Conv\left(B_{i}\right)*M_{b}.$$

Ultimately, the refinement feature (${\mathrm{RF}}_{b}$) is obtained by determining the element-wise sum of the attended feature and the 3 × 3 convolution operation for the frame $B_{i}$.

(7) $${\mathrm{RF}}_{b}=Conv\left(B_{i}\right)+A_{b}.$$

The above process is applied identically to the previously recovered sharp frame ($S_{i-1}$) to obtain another refinement feature ${\mathrm{RF}}_{s}$.

**Figure 4:**
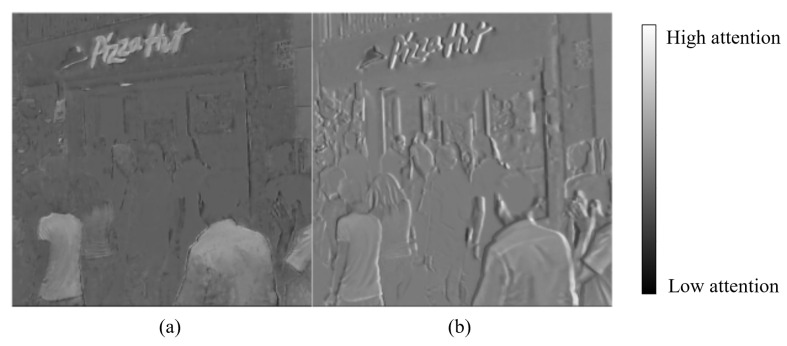
Example attention map of the RHA module. (**a**) The attention map $M_{b}$ obtained from the current blurry frame. (**b**) The attention map $M_{s}$ obtained from the previously recovered sharp frame.

An example of an attention map obtained from the operation of the RHA module is shown in Figure 4. The attention map obtained from the current blurry frame places a higher weight on certain texture regions than the edge region. In contrast, the attention map obtained from the previously recovered sharp frame places more weight on the edge region. Accordingly, the two attention maps contain complementary information, thus being useful in a refinement network that uses such information as an input.

### 3.3. Stage 2: Refinement Network

The refinement network refines the coarse frame obtained in the first stage. In particular, this network operates using frame images, unlike the coarse network, which mainly uses event data. As shown in Figure 2, the coarse frame ($C_{i}$) obtained from the coarse network and the refinement features (${\mathrm{RF}}_{b}$ and ${\mathrm{RF}}_{s}$) obtained from the RHA modules are used as an input.

Specifically, the process of creating an input for the refinement network is as follows. As explained in the previous section, each RHA module is applied to a current blurry input frame ($B_{i}$) and previously recovered sharp frame ($S_{i-1}$) to extract useful information for refinement.

(8) $${\mathrm{RF}}_{b}=RHA\left(B_{i},C_{i}\right)$$

and

(9) $${\mathrm{RF}}_{s}=RHA\left(S_{i-1},C_{i}\right),$$

where RHA() denotes the residual hint attention module described in Section 3.2.

${\mathrm{RF}}_{b}$ and ${\mathrm{RF}}_{s}$, obtained from the RHA modules, become the input feature ($F_{i}$) for the refinement network, where they are added to the coarse feature obtained from a 3 × 3 convolution operation.

(10) $$F_{i}=Conv\left(C_{i}\right)+{\mathrm{RF}}_{s}+{\mathrm{RF}}_{b}.$$

**Figure 5:**
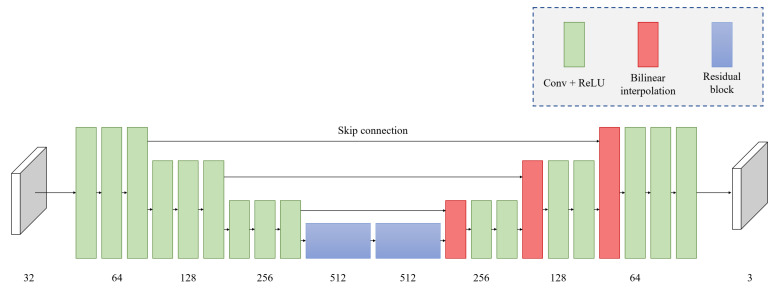
Structure of the refinement network.

As shown in Figure 5, the refinement network has an encoder–decoder structure. The encoder extracts features from input ($F_{i}$) through convolution operations on three spatial scales. On each scale, spatial downsampling is performed using one 3 × 3 convolution layer with a stride of 2 after passing through two convolution layers consisting of a 3 × 3 convolution with a stride of 1 and ReLU activation. Thereafter, the two residual layers serve the role of a bottleneck.

The decoder, consisting of convolution layers, restores the information on the three scales. On each spatial scale, after performing spatial upsampling through bilinear interpolation, two convolution layers are passed through. Each convolution layer performs 3 × 3 convolution with a stride of 1 and ReLU activation.

The layers of the encoder and decoder with the same spatial scale are connected through skip connections. If the entire process of the refinement network is expressed as RN(), the final output frame $S_{i}$ is obtained by adding the residual from the refinement network and the coarse frame $C_{i}$, as shown in the equation below:

(11) $$S_{i}=RN\left(F_{i}\right)+C_{i}.$$

### 3.4. Loss Function

The proposed MEVDNet uses two types of loss for learning: $L_{coarse}$, which is the loss for two latent sharp frames (${\mathrm{LS}}^{\left(1\right)}$, ${\mathrm{LS}}^{\left(2\right)}$) obtained from the residual estimation module of the coarse network, and $L_{refine}$, which is the loss for the final output $S_{i}$.

$L_{coarse}$ is the MSE loss between the ground truth (GT) and two latent sharp frames obtained from the current blurry frame and the previously recovered sharp frame, respectively. This can be defined as:

(12) $$L_{coarse}=\frac{1}{2HW}(∥{\mathrm{LS}}^{\left(1\right)}-\mathrm{GT}{∥}^{2}+∥{\mathrm{LS}}^{\left(2\right)}-\mathrm{GT}{∥}^{2}),$$

where *H* and *W*, respectively, denote the height and width of the frame.

$L_{refine}$ is the MSE loss between the final output obtained through the refinement network and the ground truth.

(13) $$L_{refine}=\frac{1}{HW}(∥S_{i}-\mathrm{GT}{∥}^{2}).$$

The total loss ($L_{total}$) obtained by combining the two types of loss is represented as follows:

(14) $$L_{total}=\lambda_{1}L_{coarse}+\lambda_{2}L_{refine},$$

where the parameters $\lambda_{1}$ and $\lambda_{2}$ were set to 0.01 and 1.0, respectively, in our experiments.

## 4. Experiments and Results

### 4.1. Experiment Settings

The proposed MEVDNet was trained and tested using the GoPro synthetic dataset [22] and HQF real dataset [23]. The GoPro synthetic dataset was used for frame-based video deblurring. The synthetic events were generated using the event simulator ESIM [51] in the GoPro dataset [22]. Furthermore, sharp frames were averaged to express dynamic blur frames, as used in previous studies [1,13,22,24]. In this study, blur frames were generated dynamically using 1–15 frames. The GoPro synthetic dataset consists of 33 video sequences and 4702 frames. As a result, the generated GoPro synthetic dataset tended to present a mix of severely blurred frames and weakly blurred frames. The HQF real dataset [23] consists of sharp frames and real event data captured from an event camera (DAVIS240C). The blurry frames were generated by the same method as the GoPro synthetic dataset using seven frames for the training and testing. The HQF real dataset contains 11 video sequences and 2212 frames. Compared to the GoPro synthetic dataset, the HQF real dataset has faster camera movements, resulting in the continuous appearance of severely blurred frames in the video.

The Adam optimizer was used for the learning of MEVDNet. The learning rate was fixed at 0.0001 for the first 10 epochs, and then scheduled to linearly decrease until 250 epochs. The beta values of the Adam optimizer were set to (0.9, 0.99). Furthermore, the batch size for learning was set to four, and learning was implemented with PyTorch [52]. The number of iterations was 763 for training the model with the GoPro dataset and 397 with the HQF dataset. All tasks were executed through a Nvidia Titan RTX GPU machine.

The comparative experiment employed (1) the event-to-video reconstruction model, which predicted frame information through only event data; (2) frame-based video deblurring models, which used only blurry frames as an input; and (3) event-based video deblurring models, which additionally used event data. E2VID [21] was used as the event-to-video reconstruction model, in which event data were restored to frame images using ConvLSTM [53]. A comparison with E2VID clearly demonstrated the limitations regarding texture restoration when restoration was performed using only event data without frame information. For the comparison with the frame-based video deblurring models, STFAN [5], CDVD-TSP [8], and ESTRNN [10] were used. These are deep-learning-based models that attempt to improve the picture quality of deblurred results through frame alignment as well as optical flow and recurrent techniques using CNN networks. The comparison with the frame-based video deblurring models highlighted the limitations of the methods that did not employ event data.

LEDVDI [12] and $D^{2}$Net [13] were compared for event-based video deblurring. LEDVDI is an event-main method, where the intensity residual is predicted through deep learning methods and deblurring is performed. $D^{2}$Net is a frame-main method in which high-level features of frame-based video deblurring are reweighed as event data. A comparison with LEDVDI highlighted the changes induced by two-stage refinement, whereas a comparison with $D^{2}$Net showed the difference between the event-main and frame-main methods of event-based video deblurring.

The peak signal-to-noise ratio (PSNR) and structural similarity (SSIM) metrics were used for the quantitative comparison. Note that these image quality metrics have been used to develop most of the existing methods of image and video deblurring. This is because these metrics are commonly used to objectively assess picture quality for many image manipulation tasks. Specifically, PSNR and SSIM are full-reference image quality metrics, and we had deblurred frames and ground-truth frames. Hence, we used PSNR and SSIM, which have commonly been employed in previous studies [12,13].

### 4.2. Qualitative Results

**Figure 6:**
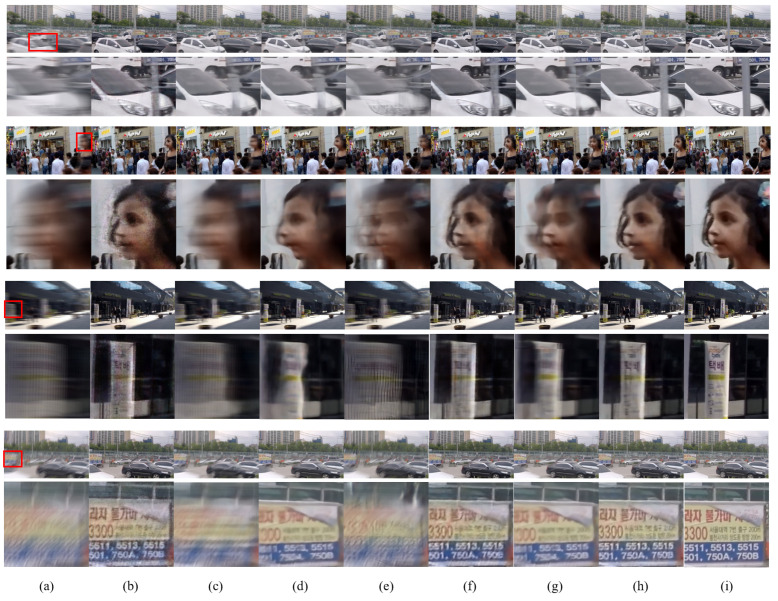
Qualitative comparison of the results using the GoPro synthetic dataset. (**a**) Input blurred image. (**b**) E2VID [21]. (**c**) STFAN [5]. (**d**) CDVD-TSP [8]. (**e**) ESTRNN [10]. (**f**) LEDVDI [12]. (**g**) $D^{2}$Net [13]. (**h**) Proposed MEVDNet. (**i**) Ground truth.

Figure 6 shows the qualitative comparison of the results obtained using the GoPro synthetic dataset [22]. After deblurring with E2VID [21], excessive noise was present in all comparison images (e.g., the stain noise on the girl’s face). The noisy regions matched the parts with a blur. Note that motion blur is generated in the region of a moving object, which is also the region of event onset. Based on this fact, it can be inferred that the acquisition noise in the event data had a direct negative impact on the output image. After deblurring with STFAN [5], CDVD-TSP [8], and ESTRNN [10], which are the frame-based video deblurring methods, slight differences were observed, but motion blur remained in all of these cases. In particular, almost no restoration was achieved in the challenging cases with severely blurred frames. Based on the visual results, we compared the methods in detail by highlighting the cases in which restoring the blurred frames was difficult.

First, slim objects such as utility poles may move at a fast speed (see the first row of images in Figure 6). In this case, the object becomes difficult to recognize in the blurred frame. Therefore, the image restoration should be carried out with the structure information from adjacent frames or auxiliary data such as event data. E2VID [21] solved this problem using event data. After deblurring with E2VID, noise was present in the texture, but the shape of the utility poles was successfully restored. The frame-based methods [5,8,10] all failed to restore the utility poles. Among the event-based methods, the event-main method (i.e., LEDVDI [12] in Figure 6f) successfully restored the utility pole. However, the edges were distorted, and noise was present in the texture to a certain extent. $D^{2}$Net [13], which is a frame-main method, failed to restore the utility pole and even generated visual artifacts (e.g., the distorted edges of the pole object). The proposed MEVDNet model, on the other hand, showed straight edges and stable textures.

Second, images of a person’s face were compared (see the second row in Figure 6). In this case, some face regions such as the eyes and nose were frequently distorted due to the severe motion blur. Despite some noise, LEDVDI [12] produced clear images. The frame-based methods [5,8,10] produced human faces that were distorted in various ways, such as through ghost artifacts. LEDVDI [12] restored all facial features to a level very similar to the ground truth. However, due to traces of the event data, the texture was smudged in the region where a blur was present. $D^{2}$Net [13] produced an incorrect structure that was severely distorted. MEVDNet adequately restored the details and produced a stable texture for the person’s face.

Third, a motion blur was input to the extent that the original image was difficult to recognize (see the third row in Figure 6). In this case, the deblurring algorithm should have been dependent on auxiliary information such as the adjacent frames or event data, since it was extremely challenging to obtain any useful information from the current blurry frame. LEDVDI [12] produced ripple noise in the billboard. With $D^{2}$Net [13], the blur was not completely removed, and visual artifacts were generated above the billboard. For example, the billboard’s edge was unnaturally misaligned.

Fourth, the effect of the deblurring methods on text images was compared (see the last row in Figure 6). E2VID [21] and the frame-based methods [5,8,10] demonstrated the same tendency as before. The text deblurred by E2VID had noise, while the frame-based methods were ineffective for restoring the text in the severely blurred frame. LEDVDI [12] successfully generated clear text, but the traces of the blur around the text created a smudged texture. $D^{2}$Net [13] produced a text image in which the blur was not removed, in contrast to the results obtained with LEDVDI and the proposed method. The proposed method removed the traces of blur present in the texture more effectively compared with LEDVDI [12]. Therefore, the latter demonstrated the best picture quality in the qualitative comparison of the results.

**Figure 7:**
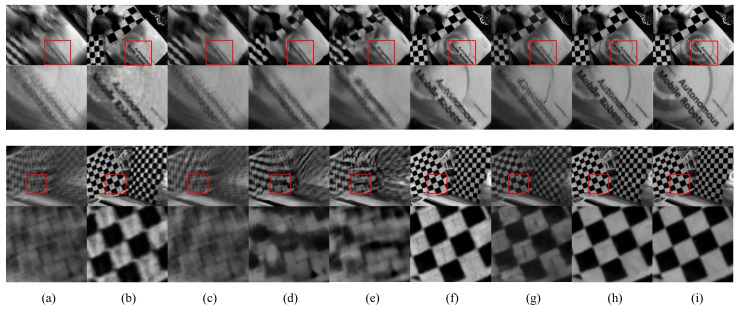
Qualitative comparison of the results using the HQF real dataset. (**a**) Input blurred image. (**b**) E2VID [21]. (**c**) STFAN [5]. (**d**) CDVD-TSP [8]. (**e**) ESTRNN [10]. (**f**) LEDVDI [12]. (**g**) $D^{2}$Net [13]. (**h**) Proposed MEVDNet. (**i**) Ground truth.

Figure 7 shows the qualitative comparison of the results obtained from the HQF dataset [23]. As seen from the figure, the proposed method showed better visual results than the existing methods. As we observed in the GoPro dataset, the proposed method provided clearer text characters and better textures than those of the existing methods. More visual results can be found in the Supplementary Materials.

### 4.3. Quantitative Results

Table 2 presents the quantitative results derived using the GoPro synthetic dataset [22]. The GoPro synthetic dataset consisted of severely blurred frames and weakly blurred frames that appeared alternately. E2VID [21] resulted in low PSNR and SSIM values because it generated noise regardless of the blur strength. The frame-based methods (i.e., STFAN [5], CDVD-TSP [8], and ESTRNN [10]) were relatively ineffective for the challenging cases with fast motion. As seen from the table, the frame-based video deblurring methods had lower PSNR and SSIM values than the event-based methods (i.e., LEDVDI [12], $D^{2}$Net [13], and the proposed method). As observed in the qualitative results, LEDVDI outperformed $D^{2}$Net for deblurring severely blurred frames, whereas LEDVDI still generated noise and visual artifacts for weakly blurred frames. Therefore, the average performance in terms of PSNR and SSIM was inferior to that of $D^{2}$Net, which is a frame-main method. The results obtained with $D^{2}$Net contrasted with those from LEDVDI, which is an event-main method. $D^{2}$Net was effective for weakly blurred videos, but the picture quality was inferior to that of LEDVDI and the proposed method for severely blurred frames, as observed in the examples of the qualitative results. $D^{2}$Net outperformed LEDVDI in terms of the average PSNR and SSIM values. These results indicated that the visual artifacts caused by the direct use of event data had a significant impact on the picture quality, even though the event-main method was capable of deblurring a stronger blur. The proposed method resulted in the highest PSNR and SSIM values among the compared models, demonstrating that the event-main method with an additional refinement stage could utilize the advantages of event data for motion restoration while mitigating the visual artifacts associated with the direct use of event data for motion deblurring.

Table 3 shows a comparison of the quantitative results using the HQF real dataset [23] for the event reconstruction [21], frame-based video deblurring [5,8,10], and event-based video deblurring [12,13] methods. The frame-based video deblurring methods, which mainly use frame information, showed a low picture quality in terms of blurring due to the fast motion of the HQF real dataset, while E2VID [21], which depends on event data, showed relatively high PSNR and SSIM values. LEDVDI [12], which is an event-based method, also showed high values due to its characteristics. In contrast, D2Net [13], which has a high dependence on frame information, showed lower PSNR and SSIM values than LEDVDI. The proposed method showed the best performance for the HQF dataset, indicating that the second stage of frame-based refinement did not negatively affect the restoration performance in terms of the picture quality, even in challenging cases.

The results implied that the proposed method could not only improve the picture quality of videos but also be helpful for extracting high-level semantic information in various video recognition tasks, such as object detection and semantic segmentation. Note that it is necessary to deblur real-world video sequences prior to the main recognition tasks when the target video has severely blurred frames. We believe that the proposed method could be used to improve recognition accuracy, particularly in challenging scenarios with blurred frames.

### 4.4. Effect of the Attention Module

In the proposed method, the RHA module was designed to extract useful information for refinement between the coarse network and the refinement network. In a previous study (MPRNet [54]), a supervised attention module (SAM) was proposed to extract useful information for a multi-stage approach while serving a similar purpose to the RHA module. The SAM generated an attention map using the output from the first stage and suppressed the features of the first stage such that only useful information was used in the second stage. In other words, it received the output of the first stage as an input, and the attention target was the feature map used for obtaining the output of the first stage. In the RHA module, the attention map was acquired using a residual hint, which was the difference between the first stage output and the frame information provided as the first input in the first stage. The attention target was frame information. The difference in the PSNR and SSIM obtained with the SAM module and the RHA module is presented in Table 4. As seen from the table, the lowest value was obtained when no attention module was present. When features were delivered to the second stage through the SAM module, higher values were obtained than in the absence of the attention module. Applying the RHA module resulted in better picture quality than applying the SAM module, possibly due to the following two reasons: (1) the use of a residual hint and (2) the effective connection between the coarse and refined stages executed in different domains (i.e., event-domain processing in the first stage and frame-domain processing in the second stage).

The residual hint refers to the difference between the coarse output frame of the first stage and the blurry input frame of the first stage. The residual hint indicates the location and extent of changes performed for deblurring in the first stage. Therefore, using the residual hint was more advantageous for extracting important information in the second stage than directly using the coarse output in predicting the attention map.

Furthermore, unlike MPRNet [54], where both the first and second stages were executed in the frame domain, the proposed method involved an event-domain task executed in the first stage and a frame-domain task executed with the coarse frame in the second stage. The SAM module performed the attention operation for the features extracted in the first stage and then delivered them to the second stage. In contrast, the RHA module performed the attention operation for the frame input added in the second stage. Specifically, the RHA module exhibited a higher restoration quality because it was designed to deliver information according to the domain processed in the second stage.

### 4.5. Effect of the Two Stages

This study proposed a refinement network that achieves the final output by applying refinement to the coarse output obtained from a coarse network. This ablation study evaluated the effects of each stage on video deblurring.

Table 5 presents the results when each stage was omitted. Without the coarse stage, the PSNR and SSIM values reflected deblurring performed only by frame-based refinement, without event-based video deblurring in the first stage. The PSNR and SSIM values both decreased, by 6.01 dB and 0.057, respectively, because the coarse stage did not involve event-based video deblurring. Without the refinement stage, the results reflected event-based video deblurring when refinement was not executed. When the entire refinement stage was absent, the PSNR and SSIM values decreased by 1.52 dB and 0.16, respectively.

### 4.6. Discussion

The proposed method is a multi-stage approach that enhances the deblurring performance of the existing event-main method through the refinement of the first-stage output. The proposed method showed superior performance in terms of picture quality for the synthetic and real event datasets compared to the existing methods. However, in an image processing task, the performance gain should be considered in terms of not only the picture quality (e.g., PSNR and SSIM), but also the computational complexity (e.g., GMAC and running time). Table 6 shows the computational complexity in terms of the GMAC and running time measured for the GoPro synthetic dataset with an image size of 1280 × 720 and the HQF real dataset with an image size of 240 × 180, respectively.

It should be noted that the proposed method achieved better picture quality in terms of PSNR and SSIM, while the computational complexity in terms of GMAC and running time increased by a relatively small amount compared with LEDVDI. Specifically, the proposed method improved the picture quality in the experiment using the GoPro dataset (i.e., the differences were PSNR 1.5dB and SSIM 0.016). As mentioned in the Introduction, event data have several advantages for the video deblurring task, owing to their low latency and reduced motion blur. However, event data often contain acquisition noise and inherently lack texture information. Hence, the direct use of event data can often generate visual artifacts in the final output image (e.g., image noise and incorrect textures). As observed in Figure 6f and Figure 7f of the LEDVDI’s visual results, the visual artifacts were not trivial. To tackle this issue in event-based deblurring, we proposed a two-stage network by adding a frame-based refinement stage that utilizes all the available frames with more abundant textures to further improve the picture quality of the first-stage coarse output. Note that the video in the GoPro dataset consisted of both severely blurred frames and weakly blurred frames. Hence, we could extract more image structure and texture information from the weakly blurred frame and use it for the frame-based refinement in the second stage of our network. As a result, we observed a performance improvement in terms of picture quality (PSNR and SSIM).

However, it is also true that if all of the input frames are severely blurred frames, the event-main approach (i.e., LEDVDI) will provide better picture quality because only event data are used for deblurring. Additionally, the frame-based refinement approach used in the proposed method may negatively affect the picture quality of the final output. Note that the video in the HQF dataset consisted of only severely blurred frames caused by fast camera motions. As shown in the example images in Figure 7a, it was difficult to extract the structure and texture information from the original input frames. In this challenging case of the HQF dataset, the experimental results revealed that the proposed method could also achieve a slight improvement in the picture quality (PSNR 0.24 dB and SSIM 0.005), though the improvement was not higher than that of the GoPro dataset. More importantly, this result indicated that the proposed method was not negatively affected by the frame-based refinement, even in the challenging case with only severely blurred frames. Overall, the proposed method was better than the existing methods in terms of the picture quality, even though it increased the computational complexity by a small amount. A new method should be developed to achieve a performance improvement in terms of both picture quality and computational complexity. This remains an important avenue for future work.

Furthermore, it should also be noted that there were some limitations to this study, in that the proposed method only used one previously recovered frame rather than multiple frames recovered from previous points in the video. Therefore, as a part of any future work, it is necessary to develop an advanced algorithm that can utilize multiple frames in the event-based deblurring stage. Additionally, the proposed method performed the deblurring process using the event data aggregated along the time axis. However, since the aggregated data lost some temporal information, it was difficult to fully utilize the temporal information of the event data. Note that some previous works have used queue-based methods for better event embedding in depth estimation [17]. However, these methods are not suitable for deblurring tasks that require the preservation of event data over a long exposure time. It is inefficient to store all event data for a long period of time, since this requires a lot of memory. Therefore, a new event embedding method for video deblurring should be developed to improve picture quality. This also remains as an important avenue for future work.

## 5. Conclusions

This study proposed MEVDNet, which is a two-stage deep learning network for refining the results of event-based video deblurring. MEVDNet obtains a coarse output in which the motion blur is primarily removed by the event data in the coarse network. Thereafter, the final output frame is obtained by refining the coarse output using the frame information in the refinement network. Between the first and second stages, the RHA module is designed to extract useful information for frame-based refinement. In addition, video deblurring is performed for blurry image sequences using the previously recovered sharp frames. Qualitative and quantitative comparison experiments conducted using synthetic and real datasets revealed that the proposed method was effective in improving the picture quality of deblurred results. The experimental results demonstrated that event-based video deblurring maintained an outstanding picture quality even for severely blurred frames, while mitigating visual artifacts such as unstable textures and distorted edges. The ablation study also proved that the proposed frame-based refinement and RHA module were effective in terms of the picture quality of deblurred frames. Overall, this study verified the feasibility of an event-based deep learning model that uses a two-stage coarse-refinement structure as well as an RHA module.

## Figures and Tables

**Table 1:** Summary of related works on video deblurring.

Method		Description
Frame-based video deblurring	STFAN [5]	Frame-based deblurring method that performs the alignment using a kernel prediction network.
	CDVD-TSP [8]	Frame-based deblurring method that utilizes the adjacent frames aligned with the optical flow in an image sequence.
	ESTRNN [10]	Recurrent neural network (RNN) that performs temporal feature fusion.
Event-based video deblurring	EDI [20]	Handcrafted method using the event-main approach that directly computes the residual frame from event data.
	LEDVDI [12]	CNN model of the event-main approach that predicts the residual frame from event data.
	$D^{2}$Net [13]	CNN model of the frame-main approach that uses a frame-based deblurring framework and fuses the image features with event features extracted from event data.

**Table 2:** Quantitative results of video deblurring using the GoPro synthetic dataset.

	E2VID [21]	STFAN [5]	CDVD-TSP [8]	ESTRNN [10]	LEDVDI [12]	$D^{2}$Net [13]	MEVDNet
PSNR	31.06	31.60	33.76	33.51	35.14	35.69	36.66
SSIM	0.904	0.900	0.925	0.912	0.941	0.950	0.957

**Table 3:** Quantitative results of video deblurring using the HQF real dataset.

	E2VID [21]	STFAN [5]	CDVD-TSP [8]	ESTRNN [10]	LEDVDI [12]	$D^{2}$Net [13]	MEVDNet
PSNR	27.54	22.48	22.39	23.23	30.77	29.33	31.01
SSIM	0.829	0.679	0.694	0.688	0.905	0.880	0.910

**Table 4:** Effect of the proposed attention module (RHA).

	No Attention Module	SAM	RHA
PSNR	30.65	35.14	36.66
SSIM	0.900	0.941	0.957

**Table 5:** Effect of the two stages.

	Without Coarse Stage	Without Refinement Stage	Our Model
PSNR	30.65	35.14	36.66
SSIM	0.900	0.941	0.957

**Table 6:** GMAC and running time for the GoPro [22] and HQF datasets [23].

	GoPro		HQF	
	**GMAC**	**Running Time (s)**	**GMAC**	**Running Time (s)**
E2VID [21]	430.58	0.14	20.63	0.009
STFAN [5]	613.82	0.24	29.41	0.016
CDVD-TSP [8]	5123.91	1.78	244.73	0.171
ESTRNN [10]	1772.07	0.54	83.07	0.084
LEDVDI [12]	1219.42	0.30	57.16	0.019
$D^{2}$Net [13]	5200.76	1.99	248.33	0.298
MEVDNet	1710.26	0.39	80.17	0.023

## Data Availability

Not applicable.

## Supplementary Materials

The following supporting information can be downloaded at: https://www.mdpi.com/article/10.3390/s23062880/s1.
