# Supplementary material for: Multi-Stage Network for Event-Based Video Deblurring with Residual Hint Attention
Source: Sensors (Basel). 2023 Mar 7;23(6):2880. doi: 10.3390/s23062880 (PMC10056412; doi:10.3390/s23062880)
Supplement: Supplementary file 1 [file sensors-23-02880-s001.zip › sensors-2212416-supplementary.pdf]

Supplementary Materials: Multi-Stage Network for Event-Based Video Deblurring with Residual Hint Attention

Jeong Min Kim and Yong Ju Jung*

1. Supplementary details

This supplementary material is accompanied by the manuscript "Multi-stage network for event-based video deblurring with residual hint attention" and contains the network architectures and additional visual results.

Table S1 shows the network architecture of the coarse network. Table S2 shows the structure of the dynamic filter estimation. Table S3 shows the structure of the gate fusion module. Table S4 shows the structure of the residual hint attention (RHA) module. Table S5 shows how the inputs of the refinement network are generated. Table S6 shows the network architecture of the refinement network.

Figure S1 shows additional results of visual comparison with the GoPro dataset [1].

References

1. Nah, S.; Kim, T.H.; Lee, K.M. Deep multi-scale convolutional neural network for dynamic scene deblurring. In Proceedings of the IEEE Conference on Computer Vision and Pattern Recognition, 2017, pp. 3883–3891.
2. Lin, S.; Zhang, J.; Pan, J.; Jiang, Z.; Zou, D.; Wang, Y.; Chen, J.; Ren, J. Learning event-driven video deblurring and interpolation. In Proceedings of the European Conference on Computer Vision. Springer, 2020, pp. 695–710.
3. Zhou, S.; Zhang, J.; Pan, J.; Xie, H.; Zuo, W.; Ren, J. Spatio-temporal filter adaptive network for video deblurring. In Proceedings of the IEEE/CVF International Conference on Computer Vision, 2019, pp. 2482–2491.
4. Rebecq, H.; Ranftl, R.; Koltun, V.; Scaramuzza, D. High speed and high dynamic range video with an event camera. *IEEE Transactions on Pattern Analysis and Machine Intelligence* **2019**, *43*, 1964–1980.
5. Pan, J.; Bai, H.; Tang, J. Cascaded deep video deblurring using temporal sharpness prior. In Proceedings of the IEEE Conference on Computer Vision and Pattern Recognition, 2020, pp. 3043–3051.
6. Zhong, Z.; Gao, Y.; Zheng, Y.; Zheng, B. Efficient spatio-temporal recurrent neural network for video deblurring. In Proceedings of the European Conference on Computer Vision. Springer, 2020, pp. 191–207.
7. Shang, W.; Ren, D.; Zou, D.; Ren, J.S.; Luo, P.; Zuo, W. Bringing events into video deblurring with non-consecutively blurry frames. In Proceedings of the IEEE/CVF International Conference on Computer Vision, 2021, pp. 4531–4540.

Table S1. Structure of the coarse network [2].

Inputs/Branches	Operation	Kernel Size	Stride	Channel	Output	Activation
Input	Concatenation (C_{i-1}, C_i)					
E_encoder1	Convolution	3×3	1	64	e_feature1	LeakyReLU
E_resblock1_1	Residual block	3×3	1	64	e_feature2	LeakyReLU
E_resblock1_2	Residual block	3×3	1	64	e_feature3	LeakyReLU
E_encoder2	Convolution	3×3	2	96	e_feature4	LeakyReLU
E_resblock2_1	Residual block	3×3	1	96	e_feature5	LeakyReLU
E_resblock2_2	Residual block	3×3	1	96	e_feature6	LeakyReLU
E_encoder3	Convolution	3×3	2	128	e_feature7	LeakyReLU
E_resblock3_1	Residual block	3×3	1	128	e_feature8	LeakyReLU
E_resblock3_2	Residual block	3×3	1	128	e_feature9	LeakyReLU
kconv	kconv(e_feaeture9, Dynamic Filter), 128channel					
E_decoder1	ConvTranspose	4×4	2	96	e_feature10	LeakyReLU
Skip connection	Concatenation(e_feature10, e_feature6)					
E_skipConv1	Convolution	3×3	1	96	e_feature11	LeakyReLU
E_resblock4_1	Residual block	3×3	1	96	e_feature12	LeakyReLU
E_resblock4_2	Residual block	3×3	1	96	e_feature13	LeakyReLU
E_decoder2	ConvTranspose	4×4	2	64	e_feature14	LeakyReLU
Skip connection	Concatenation(e_feature13, e_feature3)					
E_skipConv2	Convolution	3×3	1	96	event feature(E_i)	LeakyReLU
residual_estimation_for_blurry_frame	event feature(E_i)					
E_residual_conv_b1	Convolution	3×3	1	32	r_b_feature1	LeakyReLU
E_residual_resblock_b1	Residual block	3×3	1	32	r_b_feature2	LeakyReLU
E_residual_resblock_b2	Residual block	3×3	1	32	r_b_feature3	LeakyReLU
E_residual_conv_b2	Convolution	3×3	1	3	intensity_residual_b	LeakyReLU
	$(B_i * intensity\_residual\_b = LS_i^{(b)})$					
residual_estimation_for_previous_frame	event feature(E_i)					
E_residual_conv_s1	Convolution	3×3	1	32	r_s_feature1	LeakyReLU
E_residual_resblock_s1	Residual block	3×3	1	32	r_s_feature2	LeakyReLU
E_residual_resblock_s2	Residual block	3×3	1	32	r_s_feature3	LeakyReLU
E_residual_conv_s2	Convolution	3×3	1	3	intensity_residual_s	LeakyReLU
	$(S_{i-1} * intensity\_residual\_s = LS_i^{(s)})$					
Gate_fusion	$(C_i, C_{i-1}, LS_i^{(b)}, LS_i^{(s)}, PS_i^{(2)})$					
	$(CS_i = weight1 * LS_i^{(b)} + weight2 * LS_i^{(s)})$					
Output	coarse sharp frame (CS_i)					

Table S2. Structure of the dynamic filter generation block used for the event feature extraction [2,3].

Inputs/Branches	Operation	Kernel Size	Stride	Channel	Output	Activation
Input	Concatenation(C_{i-1}, C_i, B_{i-1}, B_i, S_{i-1})					
F_encoder1	Convolution	3×3	1	64	f_feature1	LeakyReLU
F_resblock1_1	Residual block	3×3	1	64	f_feature2	LeakyReLU
F_resblock1_2	Residual block	3×3	1	64	f_feature3	LeakyReLU
F_encoder2	Convolution	3×3	2	96	f_feature4	LeakyReLU
F_resblock2_1	Residual block	3×3	1	96	f_feature5	LeakyReLU
F_resblock2_2	Residual block	3×3	1	96	f_feature6	LeakyReLU
F_encoder3	Convolution	3×3	2	128	f_feature7	LeakyReLU
F_resblock3_1	Residual block	3×3	1	128	f_feature8	LeakyReLU
F_resblock3_2	Residual block	3×3	1	128	f_feature9	LeakyReLU
FAC_conv1	Convolution	3×3	1	128	f_feature10	LeakyReLU
F_resblock4_1	Residual block	3×3	1	128	f_feature11	LeakyReLU
F_resblock4_2	Residual block	3×3	1	128	f_feature12	LeakyReLU
FAC_conv2	Convolution	3×3	1	128×K×K	Dynamic Filter	LeakyReLU
Output	Dynamic Filter (128×K×K)					

Table S3. Structure of the gate fusion module

Inputs/Branches	Operation	Kernel Size	Stride	Channel	Output	Activation
Input	$C_i, C_{i-1}, S_{i-1}, LS_i^{(b)}, LS_i^{(s)}$					
Reshape	reshape each input.					
Input	Concatenation(reshape($C_i, C_{i-1}, S_{i-1}, fPS_i^{(1)}, PS_i^{(2)}$))					
G_conv3d1	Convolution 3D	3×3	1	64	gate_feature1	LeakyReLU
G_conv3d2	Convolution 3D	3×3	1	64	gate_feature2	LeakyReLU
G_conv3d3	Convolution 3D	3×3	1	6	weight	sigmoid
Split	split weight to 3 channel $weight1 = weight[:, :3, ...], weight2 = weight[:, 3:, ...]$					
Output	Two channel weight map ($weight1, weight2$)					

Table S4. Structure of the residual hint attention (RHA) module

Inputs/Branches	Operation	Kernel Size	Stride	Channel	Output	Activation
Input			(CS_i, frame)			
			$ResidualHint = frame - CS_i$			
A_att_conv	Convolution	3×3	1	32	attention_map	sigmoid
Input			(frame)			
A_content_conv	Convolution	3×3	1	32	content_feature	LeakyReLu
Input			(frame)			
A_ref_conv	Convolution	3×3	1	32	ref_feature	LeakyReLu
			$Attended\_feature = attention\_map * ref\_feature$			
Output			$(Attended\_feature + content\_feature)$			

Table S5. Generation of the input data for the refinement network

Inputs/Branches	Operation	Kernel Size	Stride	Channel	Output	Activation
Input			(CS_i, B_i, S_{i-1})			
Input			(CS_i)			
I_conv1	Convolution	3×3	1	32	coarse_feature	ReLU
Input			(S_{i-1})			
RHA_prev	Residual hint attention				attended_feature_S	
Input			(B_i)			
RHA_blur	Residual hint attention				attended_feature_B	
Output			$(coarse\_feature + attended\_feature\_S + attended\_feature\_B)$			

Table S6. Structure of the refinement network

Inputs/Branches	Operation	Kernel Size	Stride	Channel	Output	Activation
Input			(Feature(32 channel))			
Input			(CS_i)			
R_conv1_1	Convolution	3×3	1	64	r_feature1	ReLU
R_conv1_2	Convolution	3×3	1	64	r_feature2	ReLU
R_conv1_3	Convolution	3×3	2	128	r_feature3	ReLU
R_conv2_1	Convolution	3×3	1	128	r_feature4	ReLU
R_conv2_2	Convolution	3×3	1	128	r_feature5	ReLU
R_conv2_3	Convolution	3×3	2	256	r_feature6	ReLU
R_conv3_1	Convolution	3×3	1	256	r_feature7	ReLU
R_conv3_2	Convolution	3×3	1	256	r_feature8	ReLU
R_conv3_3	Convolution	3×3	2	512	r_feature9	ReLU
R_resblock_1	Residual block	3×3	1	512	r_feature10	ReLU
R_resblock_2	Residual block	3×3	1	512	r_feature11	ReLU
Skip_connection			(r_feature11, r_feature9)			
			Bilinear interpolation×2			
R_conv4_1	Convolution	3×3	1	256	r_feature12	ReLU
R_conv4_2	Convolution	3×3	1	256	r_feature13	ReLU
Skip_connection			(r_feature13, r_feature6)			
			Bilinear interpolation×2			
R_conv5_1	Convolution	3×3	1	128	r_feature14	ReLU
R_conv5_2	Convolution	3×3	1	128	r_feature15	ReLU
Skip_connection			(r_feature15, r_feature3)			
			Bilinear interpolation×2			
R_conv6_1	Convolution	3×3	1	64	r_feature16	ReLU
R_conv6_2	Convolution	3×3	1	64	r_feature17	ReLU
R_out_conv	Convolution	3×3	1	3	output_of_refinement	
Output			output_of_refinement			

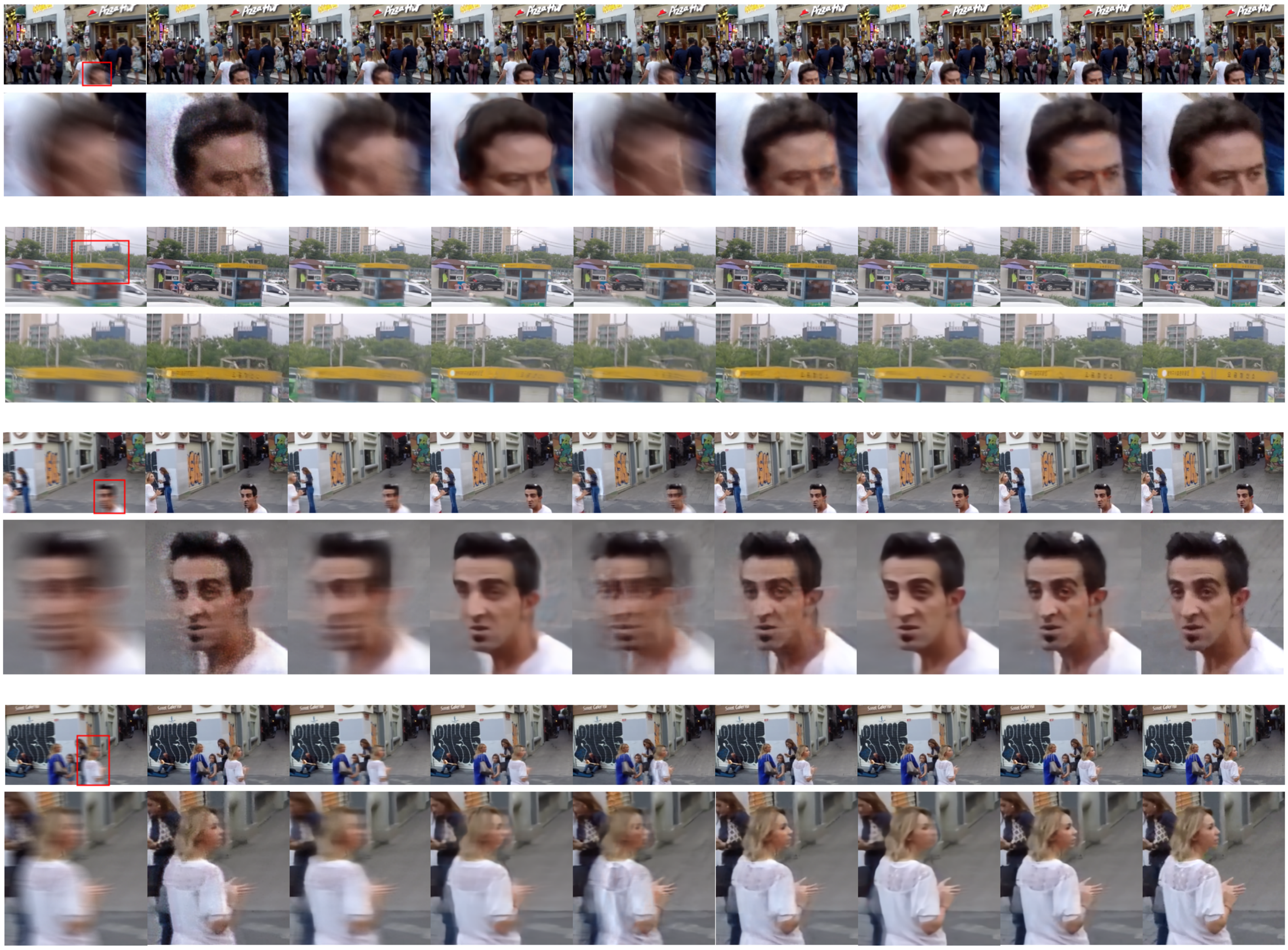

(a) (b) (c) (d) (e) (f) (g) (h) (i)

Figure S1. Additional visual comparison of video deblurring. (a) Blurry frame (b) Results of E2VID [4]. (c) Results of STFAN [3]. (d) Results of CDVD-TSP [5]. (e) Results of ESTRNN [6]. (f) Results of LEDVDI [2]. (g) Results of D^2Net [7]. (h) Results of the proposed MEVDNet. (i) Ground truth
